# Experiences of Egypt as a destination and transit country for Syrian refugee healthcare workers: a qualitative study

**DOI:** 10.1186/s12913-023-09889-4

**Published:** 2023-08-17

**Authors:** Andrew Ghobrial, Diana Rayes, Ammar Sabouni, Yamama Bdaiwi, Saad Janoudi, Natasha Howard, Aula Abbara

**Affiliations:** 1 London School of Hygiene and Tropical Medicine, London, UK; 2 Department of International Health, Johns Hopkins School of Public Health, Baltimore, MD USA; 3 Syria Public Health Network, London, UK; 4 Syria Development Centre, London, UK; 5 Department of War Studies, King’s College London, London, UK; 6 Faculty of Medicine, Kasr Al-Ainy, Cairo University, Cairo, Egypt; 7 Saw Swee Hock School of Public Health, National University of Singapore and National University Health System, Singapore, Singapore; 8 Imperial College, St Mary’s Hospital London, London, W2 1NY UK

**Keywords:** Egypt, Refugees, Doctors, Conflict, Syria, Healthcare

## Abstract

**Background:**

Refugee healthcare workers (HCWs) can make important contributions in host countries, particularly in the wake of the ongoing COVID-19 pandemic, which has exacerbated existing shortages of frontline HCWs. However, refugee HCWs often face challenges entering the labour markets of such countries even where needs exist. Syria’s decade-long conflict has forced thousands of HCWs from their homes; however, data on this population are limited, impeding the formation of policies that can support them. This study explores the experiences of Syrian refugee HCWs in Egypt.

**Methods:**

Key informants (KIs) were selected using purposive and snowball sampling method and semi-structured interviews were conducted in person in Cairo and remotely from the UK during July 2019. Interviews were conducted in Arabic and analysed using a combined deductive and inductive thematic analysis framework after transcription into English.

**Results:**

Fifteen KI interviews were analysed. The main emerging themes from the qualitative interviews are those relating to 1. Education, training, and licensing 2. Politics and bureaucracy 3. Societal factors 4. Economic factors. Political changes in Egypt altered opportunities for Syrian HCWs over time; however, refugee HCWs broadly reported acceptance among Egyptian patients and colleagues. Bureaucratic factors which impede the ability of Syrian refugee HCWs to obtain a full license to practice and leave to remain and the absence of clearly defined policies were reported as barriers. Economic factors including the risk of economic exploitation e.g. in the informal sector and financial insecurity were noted to have a negative psychosocial impact.

**Conclusions:**

This is the first qualitative research study which explores the experiences of Syrian refugee HCWs in Egypt. It adds to the sparse literature on the topic of Syrian refugee HCWs but provides evidence for further discussions on how to support refugee HCWs in Egypt and in other host countries in the region. Though interviews were conducted before the COVID-19 pandemic, the pandemic itself lends urgency to the discussion around refugee HCWs on an international level.

**Supplementary Information:**

The online version contains supplementary material available at 10.1186/s12913-023-09889-4.

## Introduction

The COVID-19 pandemic has renewed interest in the potential contributions of refugee healthcare workers (HCWs) globally, however literature that explores their experiences of entering the workforces of transit or destination countries is sparse. Among those who have been forcibly displaced, the exact number of HCWs is unknown despite attempts to form estimates [1, 2]. However, there remains an urgent need to understand the challenges which refugee HCWs face, with a view to forming policies and initiatives to support them, particularly at a time when COVID-19 has decimated the workforce of many countries [3].

Syrians form the largest group of forcibly displaced people internationally with an estimated 6.7 million internally displaced people (IDPs), and 6.2 million as refugees; the latter primarily reside in surrounding countries with sizeable populations in Turkey (3.7 million), Lebanon (830,000 registered; 1.5 million estimated) and Jordan (675,000 registered; 1.4 million estimated) [4]. An estimated 250,000 (approximately 130,000 registered) Syrian refugees are in Egypt [5]. Despite recent political rhetoric, such as the Lebanese government’s plans to repatriate Syrian refugees [6], the likelihood of safe return for Syrians remains distant [7].

Syrians in Egypt make up more than half of Egypt’s registered refugee population. Syrians are not restricted to residing in settlements in Egypt, unlike in many neighbouring countries and so live predominantly in urban or peri-urban settings such as Greater Cairo, Alexandria, and Damietta among Egyptian nationals [8]. They are therefore less visible to provide targeted services for, and many live in poverty within these already struggling communities [9]. Since 2012, Syrians have been eligible for government-funded primary healthcare services, but despite numerous recent NGO (non-governmental organisation) and UNHCR initiatives to provide affordable healthcare to Syrians [10], access to services remains poor, especially among vulnerable groups [11]. As a result, they had access to free healthcare and were permitted to live in cities, unlike Lebanon and Turkey [12]. The political landscape in Egypt has influenced shifting societal attitudes and policies towards Syrian refugees. Such trends can be divided chronologically into: before 2012, up to the Egyptian presidential election; 2012-2013, as the Syrian conflict escalated; and 2014 onwards, as the military government in Egypt took power [13].

However, bureaucratic barriers remain among the greatest challenges for refugee HCWs, including Syrians when trying to enter the labour market of their host country. These have been noted among Syria refugee doctors elsewhere including in Europe. Germany, which hosts over 130,000 Syrian refugees, was an increasingly popular destination for Syrian refugee or migrant HCWs [14]. Though the process by which Syrian doctors retrain in Germany was clearer than in Egypt, there are multiple delays and bureaucratic barriers at the different stages of registration and retraining [14]. In Germany, there was also the added challenge of a federal system unlike Egypt and the other countries where Syrian refugee doctors reside. The slowness of the process of registration in Germany, as in Egypt, places not only a financial strain but also a mental health strain on the HCWs [15]. In Egypt, unlike in Germany, provisional registration requires further unpaid training, and prohibits Syrians from working whereas in Germany, limited registration allows doctors to work and earn [14]. In Egypt, as in Lebanon and Jordan [16], Syrian doctors may therefore have little option but to work informally to earn for their families. [13, 17].

Though there has been some limited exploration of the issues facing Syrian refugee healthcare workers in Lebanon, Turkey and Germany [18, 19], there has been virtually no discussion of the experiences of Syrian HCWs in Egypt and their access to the labour market in the literature. Restrictive labour policies in Egypt prevent highly skilled professionals from Syria to operate legally, leading to instances of informal work among Syrian HCWs. In some cases, this has led to the detention or intimidation of Syrian HCWs working informally in Egypt, affecting their access to livelihoods and job security. This research applies qualitative methodologies to explore these experiences as well as the views of Egyptian nationals who may train or encounter Syrian HCWs, and Syrian HCWs in the US, UK and Germany who used Egypt as a transit country for studying or training before reaching their destination country. This work will help to understand some of the key challenges they face, support advocacy for refugee healthcare workers, develop community resilience frameworks and inform more equitable social policies for refugee HCWs accessing the labour market in the region.

## Methods

### Key informant selection

Key informants (KIs) were contacted using a purposive snowball sampling method through HCWs already known to the authors through previous related research. They were contacted to discuss the project’s objectives and invite their participation. The recruited KIs subsequently contacted further potential candidates who they were acquainted with. AG also attended the Qasr Al Ainy Hospital in Cairo and disseminated information about the research among the resident doctors, but the one eligible participant identified did not respond further. All participants who responded to invitation were consented and interviewed, with the exception of one respondent who was unable to participate due to time constraints. 4 male Syrian HCWs and 3 NGO workers did not respond. All female HCWs contacted responded and were recruited. Recruitment stopped when there were no further responses to invitations. The criteria for eligibility as a KI for the study were: age 18 years and over; Syrian nationals who studied, trained, or worked in healthcare in Egypt; Egyptian nationals who were involved in the training, recruitment, or advocacy for Syrian HCWs living in Egypt. All candidates were contacted by telephone, WhatsApp or email and received a participant information sheet (PIS) and a consent form digitally in both English and Arabic. Candidates were asked to read through the information and consent form and ask any questions about the project before responding.

### Interviews

Two semi-structured interview guides were prepared; one for Syrian refugee KIs and another for KIs involved with training, recruitment, or advocacy for Syrian HCWs in Egypt. The interview guides were prepared with guidance from Rubin and Rubin [20] on developing a *“conversational partnership”* with research participants. The interview guides were amended after practice interviews in both English and Arabic to ensure translational coherence, and the final version was reviewed with a second native Arabic speaker. The interview guides are provided in Additional file 1.

KI recruitment for the study commenced following Institutional Review Board (IRB) approval and took place in Egypt between 12th and 25th July 2019. The interviews took place in the districts of Downtown Cairo, 6th October, Faisal, Dokki and Nasr City, at the convenience of the KIs. These ranged in location from the university hospital campuses and NGO offices to coffee shops, KI homes and co-working spaces. All interviews were conducted in Arabic by one interviewer (AG), who is a male British Egyptian and a native English and Arabic speaker. All face-to-face interviews were recorded using a Dictaphone, and all remote interviews were conducted and recorded using Skype, with the exception of one NGO worker who requested that interview notes were made rather than record their voice. All interviews were downloaded to a secure computer. Interviews were transcribed directly by AG from Arabic audio to English text on playback. The transcripts were checked by another native Arabic speaker for quality and accuracy.

### Analysis methods

A thematic analysis of the interview transcripts was used to draw together conclusions and produce a final report [21]. The thematic analysis was conducted by the interviewer using a primarily deductive approach by applying Braun and Clarke’s six-phase framework [22]. Initial themes were developed based on the literature review and the document analysis. After familiarisation with the data, the initial themes were then adapted, and NVivo 12 was used for a line-by-line coding process using open codes that were modified as a model for the developing themes, coding with a focus on the research question. While working through the data, an inductive approach was also used to incorporate new themes into the model. After all interviews were coded, the themes were combined, divided, and reordered by AG and AA to account for overlaps and cross-cutting themes until a final model was decided. This was then agreed with co-authors.

### Consent

All KIs were provided with an information sheet outlining the study and what to expect during the interview. It also outlined the IRB approval process and what would happen to their data. All KIs were advised that they could withdraw from the study at any time without giving a reason, and that they were not expected to do or say anything that put them at risk. KIs consented to the audio recording of the interviews using a dictaphone, or if over Skype through the bilaterally visible call recording feature, and the recording was stopped at any time at their request.

### Data management

The secure computer used for the study complied with LSHTM ethical standards. No sensitive data were collected from KIs, with the exception of their real name and a contact number or email address for the sole purpose of contacting them prior to or after the interview to clarify responses. The consent forms were emailed and returned by the KI digitally signed, or hard copies were signed in person. These were scanned and with the digitally signed forms, stored on a secure computer, with the hard copies destroyed in compliance with LSHTM data standards. Audio recordings were stored on a secured computer until transcription was completed and then deleted. KIs provided a pseudonym at the start of each audio recording to avoid any identifiable information being recorded. If KIs disclosed sensitive information, including names of individuals or organisations, this was redacted during transcription. The audio files were transcribed on a secure computer and the final transcript data has been stored on a secure server for use in future research for up to 10 years. KIs were allocated a reference number e.g. (R1) for direct quotation in the text due to inconsistency in the provided pseudonyms.

### Results: interview data

Interviews were conducted with 16 key informants, 11 of whom were Syrian HCWs and the remaining 5 representing NGOs advocating for refugees in Egypt: Syria Al Gad, PSTC and UNFPA Egypt. The NGO workers were a mixture of Syrian, Egyptian and other nationalities. 10 interviews were conducted face to face in Cairo between 12^th^ and 15^th^ July 20219; 1 face-to-face interview took place in the UK, and four were conducted remotely over Skype with KIs who lived in the UK, Canada, and Poland. The interviews lasted between thirty minutes and two hours. One recording file (R15) was corrupted on the device at the point of upload and was therefore not transcribed or included in the analysis. AG was unable to reschedule a repeat interview remotely due to the participants’ time constraints and therefore this interview was abandoned, resulting in 15 interviews being analysed. Table 1 details the demographic information of the study participants. Their ages have been provided in a range to preserve anonymity.

**Table 1:** Key informant demographics and location of interview. The health workers were Syrian whereas the NGO managers/ analysts were non-Syrian including Egyptian or other nationalities (latter not specified in the interviews)

**KI No**	**Gender**	**Age range**	**Profession**	**Location**	**Interview type**
R1	Male	25-34	Junior doctor (Syrian)	United Kingdom	Remote
R2	Male	25-34	Medical graduate (Syrian)	Egypt	In person
R3	Male	25-34	Resident/ fellow (Syrian)	Egypt	In person
R4	Male	55-64	Retired consultant physician (Syrian)	Egypt	In person
R5	Female	25-34	Medical graduate (Syrian)	Egypt	In person
R6	Male	25-34	Resident/ fellow (Syrian)	Egypt	In person
R7	Male	35-44	NGO medical manager	Egypt	In person
R8	Female	25-34	NGO manager	Egypt	In person
R9	Male	25-34	Medical graduate (Syrian)	Egypt	In person
R10	Female	35-44	NGO analyst	United Kingdom	Remote
R11	Male	35-44	NGO Manager	Egypt	In person
R12	Male	25-34	NGO Manager	Egypt	In person
R13	Male	25-34	Medical graduate (Syrian)	Poland	Remote
R14	Female	25-34	Medical graduate (Syrian)	Canada	Remote
R15	Male	25-34	Resident/ fellow (Syrian)	Egypt	In person
R16	Male	25-34	Dentist (Syrian)	United Kingdom	In person

*NGO* Non-governmental organisation

### Thematic analysis

**Fig. 1:**
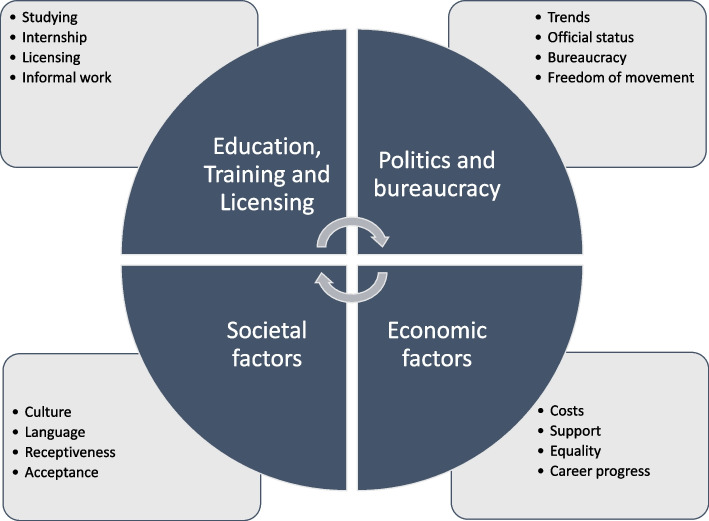
This figure shows the four main themes which emerged from the interviews from the key informants

Four main themes emerged which capture the barriers and opportunities which the KIs described. A graphic representation of the model is shown in Fig. 1.

#### Theme 1: education, training, and licensing

Nine of the HCW KIs in this study completed their medical education in Egypt after leaving Syria. Two started their first year of medical school in Egypt prior to the conflict, and the remaining seven transferred from Syria during their studies after the conflict began. The high acceptance rate for transfers from Syria and its proximity was the primary reason for coming to Egypt.

Many of the KIs felt that being allowed to continue their studies was a sufficient level of support despite the universities not offering personal or psychological support outside of academic affairs. *“I can’t really ask much more of them than what they did. They accepted us without fees like the Egyptians, so we’re grateful to them.” [R9]*

KIs report that an internship year at a government hospital is mandatory prior to finishing medical school. They note that small financial subsidies are provided to Egyptian students but not to foreign students, including Syrians, who are not paid and instead pay fees to complete the internship year. It is intended as a year to obtain the practical skills to practise medicine, but some KIs found the experience limited.* “I couldn’t even imagine that when I graduated, I wouldn’t have the capability to open a practice. It is not just a legal issue, but an issue of experience.” [R5]*

With regards to licensing, medical practice is tightly regulated by the Egyptian Medical Syndicate (EMS) and the Ministry of Health and Population (MoHP). The EMS outlines a list of requirements for Egyptian and non-Egyptian doctors [23]. Crucially, this demonstrates a pathway for foreign doctors to obtain a full licence to practise. However, the KIs unanimously expressed that there are no Syrian doctors known to have succeeded in obtaining licences after 2012. The majority of KIs who described the process identified the security approval as the point at which their applications were rejected. This is not the case for most other non-Egyptians, as Lebanese, Iraqi and Jordanian applicants known to KIs had succeeded in obtaining licences. Two KIs reported that, like Syrians, Sudanese and some Palestinian applicants also appear to have their applications rejected without any specified reason. One KI who had practised as a consultant physician in Syria for 24 years left Syria in 2012. He was offered a 3-month renewable licence to practise after speaking with the EMS.

*“This permission to work [is] essentially a kind of help for the Syrians … So they came out with this decision imagining that after a period of time we would leave. After about 3 months we’d go back [to Syria]. You know? So those were the initial ideas when we came to Egypt … but after that this situation ended … have I heard of any Syrian physician who has been able to get [a licence]? No. Not even a single one.” [R4]*

There is a lack of any official acknowledgement that Syrians cannot obtain licences however, KIs noted this to be the case in practice. Some KIs believed that it stemmed from concerns that Syrian doctors would saturate the labour market, leaving fewer jobs for Egyptians, in addition to fears that Syrians would outcompete Egyptian doctors in neighbouring practices by offering care at a lower price to refugees. One KI described a dispute between two practices due to this issue:

*“There are [Syrian] doctors … who ran a medical centre … Their pricing was reasonable. So patient check-ups were just nominal … to help Syrians. But when our Egyptian brothers realised there was a surgery with these prices, the Egyptians and Syrians started going back to this surgery. And this impacts on the Egyptian doctors … So, they reported them, and they closed the centre, imprisoned the doctors and imprisoned the Egyptian doctor who owned the centre.” [R4]*

Due to inevitable rejection and the lack of transparency surrounding the process, many Syrians are put off applying for licences and instead make plans to travel abroad following their internship year. According to KIs, Germany was the most preferred choice because of the clear process for professional registration.

KIs report that the only means for Syrian doctors to earn money from clinical practice is through informal work as all medical centres in Egypt must be registered by an Egyptian doctor who takes overall responsibility for all the clinical decisions any informal workers make. Four out of the eleven Syrian clinicians interviewed have taken up informal work. KIs note that such opportunities are usually found in under-resourced regions peripheral Cairo, where fewer MoHP inspections are carried out and the opportunities are advertised by word of mouth or social media. Syrians are recruited on the grounds of cheap labour and the employer typically turns a blind eye to the legality of their employment. In these circumstances Syrians have no working rights. Despite this, a general sense of mutual understanding was evident between the various participants. Syrian KIs speak about acknowledging the recruitment challenges in these centres and sympathise with the service managers’ approach in ensuring the facility can run. One KI described working with service managers who are unaware that Syrians cannot obtain a licence to practise. Fear of prosecution was the primary reason KIs resigned from informal work and either applied for the fellowship, found an alternative career path, or made plans to go abroad.

*“I didn’t know if [the manager] knew or not … So, when she came and asked me, I told her I didn’t have [a license to practice]. But I’d been working with her for some time, and she liked me, so she said to me that we just need to be careful. She didn’t tell me to leave but I felt that she was worried … and whenever I work privately, I feel worried about this issue.” [R5]*

Regarding other healthcare professionals, KIs from an NGO that managed medical centres around Cairo explained that because of the responsibility doctors take in decision making, they face stricter regulations compared to nursing staff. Although nurses are expected to have an official registration in Egypt, Syrian nurses can be employed much more straightforwardly and can come across as healthcare assistants during inspections. None of the KIs were particularly aware of the circumstances surrounding other allied health professionals.

#### Theme 2: politics and bureaucracy

The receptivity towards Syrian refugees in Egypt has fluctuated with political trends over time and is reflected in governmental initiatives and the general population’s attitudes towards Syrians in the country.

*“The media was swaying people … after the media was stirring about Syrians, the population started to be a bit hostile towards Syrians.” [R6]*

All KIs referred to the unpredictably shifting political and civil climate, mostly by referencing who was in power in Egypt at the time or by stating the year they are narrating and drawing parallels with the degree of bureaucracy they faced to mirror the political events.

*“That period when Morsi was in power, they opened facilitations a lot. I don’t know if [it was] because of politics or out of sympathy for Syrians, but all the Syrians benefited. Things were very straightforward … Procedures, operations were more facilitated. You’d come into the country without a visa, the treatment was just like it is for Egyptians.” [R6]*

Two Syrian KIs had been in Egypt since 2007 and noticed a shift in facilitating the entry of Syrians between 2012 and 2013. Many Syrians did not have official status and according to the KIs, there did not appear to be much enforcement at the Egyptian border. They commented on the transience of these transfer initiatives, as after 2013 university fees were reintroduced and some students were asked to pay the fees that they were exempt from for the previous year. One KI commented on the difference in treatment by university staff in the years these trends were shifting:

*“In the years after [2013], [appeals] became an impossible feat. I’m not speaking about everyone. I’m just talking about my Syrian peers. So, was this change because we initially had preferential treatment and then became just like everyone else? Or was it that we were treated normally and then it became bad treatment? I don’t know … and not that the overall sentiment was bad … you didn’t feel that other people hated you, no. But you didn’t feel as loved as before.” [R1]*

The most recurring topic raised by KIs after licensing and training was obtaining official status in Egypt. KIs noted that prior to the conflict, Syrians, like most Arab nationals, were able to apply for a tourist visa on arrival at the border. However, KIs noted this has changed with one KI noting:

*“The visa issue started in 2013 when the coup happened … There was media coverage that the Syrians were helping the party who were against the military. And this issue was broadcast in the media. So, at the time, any Syrian without leave to remain in Egypt was banned from entering Egypt, unless they had a visa. And when you went to apply for a visa, they would generally refuse them. And this happened to a lot of students who hadn’t taken leave to remain and were abroad for a visit or something … They couldn’t go back to Egypt for a year and some of them had to pay bribes of up to 3,000 dollars to be able to get back in.” [R13]*

One KI described a *“vicious cycle”* where the requirement for a valid leave to remain is needed to apply to the EMS and MoHP for a license to practice. However, a tourist visa or refugee status are not accepted as leave to remain is only granted after demonstrating evidence of current education in Egypt, employment or international entrepreneurship. KIs expressed fear of being caught by the police, as remaining with no visa resulted in financial penalties, detention or deportation.

*“There were patrols in the streets, targeting Syrians and so on* [in 2013].* So there used to be a lot of crowds at the Mogamaa and at Tahrir so they could take out their [UN] yellow cards.” [R13]*

Syrians in Egypt do not require a Refugee Status Determination Interview (RSDI) to be registered Persons of Concern (POCs) by UNHCR. However, KIs were divided about potential benefits to approaching UNHCR to register and many expressed scepticism that they would be offered financial or legal support.

*“You have no rights in the first place. So, if you went and registered as a refugee, what rights would you get? There aren’t any.” [R6]*

The biggest deterrent to registering with UNHCR was the restriction of movement in and out of Egypt. Many KIs preferred to apply for tourist visas, which required renewal every 6 months as they felt that this gave them more scope for their circumstances to change. For a time, Syrian passports were renewed every six months, and applications from refugees were assessed by a Syrian intelligence agency to force people to return for military service if they had avoided conscription by leaving the country. These rules were lifted in 2015 [24], but prior to this, many Syrians were unable to renew their passports and remained unregistered. One KI explained how he prolonged his internship year to keep his student status and avoid renewing his Syrian passport. This allowed him to visit his family and avoid military service.

*“Refugee status in Egypt traps you there. You can’t leave at all and come back. So, when I had a [student] leave to remain in Egypt and there was an opportunity to see my mother, for example. I wanted to visit her and go back to Egypt … but to be a refugee in Egypt means that I can’t leave Egypt.” [R13]*

With their families separated by the conflict, many KIs expressed difficulty trying to reunite them, even for a short time, which had an immense impact on their psychological wellbeing.

*“For me personally, I haven’t seen my mother for a year and eight months … It’s too much. It’s the longest time in my life I haven’t seen my parents… It all affects [one’s] psychology … It causes lots of other problems.” [R2]*

There was a clear sense of frustration among Syrians that the administrative processes were not transparent and lacked accountability. One KI outlined an arduous appeals process on receiving a rejected student visa application, which took one year to resolve. He was unable to find any representative who understood the process enough to advise him. The majority of KIs expressed that the bureaucracy and restrictions that limited their feeling of legitimacy in the country, their right to employment and their freedom to see their families, was ultimately what drove the decision to leave Egypt.

*“The best two years of my life were in Egypt … and my favourite people are the Egyptian people. But I felt it was impossible for me to live the rest of my life there.” [R15]*

However, leaving the country also posed difficulties as many destinations were inaccessible for similar reasons. Two KIs attempted to escape Egypt through smugglers and seek asylum on European shores.

*“I’d applied for 14 different visas outside of Egypt to continue my studies elsewhere. They were all rejected. I was rejected for visas for Germany, France, America, Korea, Turkey, China. Even China rejected me!” [R13}*

#### Theme 3: societal factors

Egyptian communities have largely embraced the Syrian population. All KIs commented on the warmth of the Egyptian public, and many would have stayed if they were able to practise their profession. One KI expressed how deeply connected he felt to Egyptians because of the treatment he experienced since arriving and was emotional about this during the interview.

*“The Egyptians are our family. To compare the treatment by the Lebanese, Jordanians or even the Turkish towards Syrians. I believe that the Egyptians treat Syrians the best … Everyone would greet me, everyone would wish me well, everyone respects me, everyone loves me. And the same goes for my family. It’s beautiful, truly.” [R4]*

The KIs felt that overall, Egyptians liked being treated by Syrian doctors and that there was very rarely any animosity in the workplace. Many commented that they could integrate themselves into Egyptian healthcare settings well, including adopting an Egyptian dialect when speaking with locals.

*“When you come as a Syrian or as another nationality, they have some interest that you’re foreign, that you’re a nice person and they’re happy to interact with you. They don’t have a bad impression of you. So if you’re nice to them they’ll carry you above their heads. I’ve had a lot of patients who would prefer to interact with me than with my peers” [R1]*

However, despite the potential for integration in Egyptian society and the bond they felt with the Egyptian people, there was a strong sense among the KIs that the limitations on their right to work inhibited the feeling of truly belonging.

*“The factors that affect the psychological state of refugee doctors … Being an outsider, that’s first. Second, is poverty … The third is the issue of the lack of ability to travel, and the lack of permission for our children to come to Egypt. The [fourth] issue is the lack of work. Work is, in truth, beneficial for a person’s psychological state … work [gives] a purpose you know, in a persons’ sense of self.” [R4]*

#### Theme 4: economic factors

Many of the psychological challenges reported by the KIs related to financial adversity. Many relied on financial support from their family who lived abroad, however depleted savings and an unstable political climate in the Middle East made this a precarious source of income. KIs expressed preferring their independence over relying on government support. There were expressions regarding the symbolism of refugee status and what they felt it represented in terms of their potential in society.

*“I refuse to seek asylum. And that’s why I was hesitant to speak with you - because I don’t want to be considered a refugee. Not because it’s offensive to be a refugee at all, not at all, but I refuse to be a refugee because I have capabilities that enable me to not be a refugee.” [R13]*

The idea of registering as a refugee was often referred to as a last resort. Some KIs had been refused financial support after registering with UNHCR, including one KI who was worried about informal work but had a two-year-old daughter.

*“We registered as refugees after we finished [medical school]. But this refugee status didn’t do anything for us to be honest … we told them we’re doctors, and we don’t have licences. So they said, ‘Even if you don’t have a licence, go and work illegally … we generally don’t give young people financial support’ … They know that we work illegally.” [R5]*

Due to the high cost of training in the Fellowship programme and its demand on their time, KIs faced a distinct choice between training and paid employment, and the need to earn for a living often presided at the expense of fulfilling career ambitions.

*“I got depressed, I don’t want this life. I don’t want to stay working just so that I can survive day to day. I want to work towards my prospects.” [R5]*

Many commented on the high costs associated with being a Syrian living in Egypt, including frequent expensive Syrian passport renewals, financial penalties for expired visas, in addition to the alarmingly high rate of inflation in Egypt,

*“Egyptians who work are dealing with inflation and can’t afford living. [Syrians are] coping with this the same as them. And then you have additional expenses. You’ve got all these things that come up: health expenses you don’t get unless you register as a refuge … You have fees for passports, leave to remain … [There’s] barely enough for living.” [R6]*

The discrepancies between the treatment of Syrians workers compared with Egyptians in the healthcare sector were noticeable, both informally and within formal training. Although KIs did not feel they were remunerated fairly for their work in the informal market, they also did not have rights to dispute their pay.

*“We started to find out that as soon as we graduated there would be differences in treatment. The Egyptian internship students earn money. They’re considered employees … But the non-Egyptian students when they do their internship, [they] pay a big sum. We paid 200 dollars for each month.” [R14]*

*“If there’s an Egyptian doctor at a practice or a medical centre, they’ll know that the Syrian doctor can’t [work legally]. So [they] won’t pay them as much as the other doctors. And obviously they aren’t treated the same as Egyptian doctors because they’re working illegally.” [R7]*

KIs reported feeling trapped by these challenges, with little scope to change their situation as all routes required some degree of financial security.

*“He can’t work in Egypt because he needs money, and he can’t travel because he needs money. He can’t work to get money, and if he gets money he doesn’t know if he can leave. I mean, it’s a really difficult situation.” [R2]*

## Discussion

This research highlights some of the experiences of Syrian HCWs in Egypt at the time of the interviews in July 2019. Though there are no policies which prohibited Syrian healthcare workers from practising after graduation alongside their Egyptian counterparts, successful entry into the workforce was noted to be very challenging in practice, particularly in the post-Morsi era after 2013. As a result of challenges, and, unlike other refugee hosting settings, Egypt was viewed as a transit country rather than a destination country for Syrian HCWs. However, since the COVID-19 pandemic escalated in early 2020, there are suggestions that there has been a change towards easing of some of the barriers, perhaps due to the significant shortages of HCWs resulting from the pandemic.

### Unofficial barriers facing Syrian HCWs in Egypt

Despite official policies which appeared to support Syrian HCWS to enter the workforce in Egypt, in practice there were multiple barriers which are noted by the KIs. The most important noted barrier to employment in healthcare in Egypt was the lack of provision of licences to practise for Syrians though, in theory they were eligible [13, 25]. However, the process was not transparent and was complicated by bureaucracy, forcing many Syrians to reluctantly take up informal work due to financial adversity. This put them at risk of financial exploitation, challenging working environments and limited training to progress their careers. Such informal work also occurred in other countries where Syrian refugee HCWs find themselves unable to work (and where they are not visible to the system), particularly in Lebanon and Jordan [16]. This can lead to financial insecurity as well as a great fear of persecution if they are caught leading to significant impacts on their mental health [15].

There are multiple barriers to legal status for Syrians in Egypt. Refusals for tourist visas since the conflict, in addition to requirements to have a visa approved prior to travel make staying in Egypt legally difficult [13]. The KIs also expressed concerns about the restrictions on financial support and on their movement out of the country to see relatives if they registered with UNHCR. For HCWs specifically, neither of these routes to obtaining a legal status in the country would allow them to work. Being granted leave to remain is the only means to obtaining a license to practice. Applicants find themselves in bureaucratic cycles of needing evidence of employment to secure leave to remain, which is needed to acquire a license to work formally.

### Psychosocial impact on Syrian refugee HCWs

All KIs note the welcome Syrians including HCWs received in Egypt, both from colleagues in healthcare and more broadly in society. This was also felt at policy level, for example the temporarily lenient medical school fees for Syrians [26]. Such experiences in Egypt, differ from Lebanon and Jordan for instance, where opportunities for undergraduate or postgraduate training or registration remain limited [27]. This contrasts with Turkey, where there are EU and Ministry of Health (MoH) initiatives which provide opportunities for Syrian HCWs, particularly doctors to work in a limited capacity in MoH facilities which provide care for Syrian refugees [28]. The draw for Syrian HCWs to Egypt is the common language shared compared to Turkey or Germany and the more welcoming environment compared to Lebanon or Jordan [16, 29].

Syrian refugee HCWs faced multiple strains on their mental health which were exacerbated by the circumstances they found themselves working in [15]. In the case of Egypt, uncertainty about their future and fear for their safety if they work informally can take its toll. Similar concerns have been raised by Syrians HCWs in Lebanon and Jordan [15, 16]. Their mental health was impacted by bureaucracy and challenges obtaining official residential status in Egypt, which restricted their ability to access work permits or apply for visas and licences for other countries. They were also unable to visit their families who often live in other countries. These restrictions lead to frustration among Syrian HCWs leaving them feeling trapped in a system without the financial means or the civil and political rights to free themselves of adversity. Similar experiences are noted among Syrian HCWs in contexts of displacement, including Germany, where bureaucratic processes are different from those in Egypt, but are also slow and difficult to navigate [14].

Importantly, the Syrian HCW KIs noted the welcome from communities and other health workers in Egypt, something which positively impacted their psychosocial health. This welcome has been noted in other refugee hosting countries including Turkey in the early years of the conflict [30]. However, as the number of refugees increased as well as rising unemployment and competition for housing or jobs, (despite the latter being restricted for refugees in many host countries) attitudes towards refugees have become increasingly negative. In part, this has been due to the protracted nature of the conflict as well as the subsequent politicization of the issue among leaders of refugee hosting countries [6, 31].

### Recommendations and opportunities to integrate Syrian HCWs in Egypt

A Global Burden of Disease study estimates a shortage of 6.4 million doctors worldwide [32]. Though the exact number of refugee HCWs is unknown, given such shortages, particularly in the wake of the ongoing COVID-19 pandemic, health systems must capitalise on the skills of those residing in their countries. Egypt is particularly affected by such shortages of doctors, with many Egyptian graduates seeking external opportunities due to a weak health system and poor opportunities to earn or train [33]. Syrian HCWs however, should opportunities exist, may be keen to remain in the region in an Arabic speaking country rather than travel to Europe or Turkey. As such, creating or enforcing existing policies which support existing Syrian HCWs to enter the health system officially are essential. Turkey’s system by which Syrian refugee HCWs can work with limited registration and limited scope to support the needs of Syrian refugees [34] is less suited to Egypt which hosts fewer Syrian refugees. Additionally, the creation of a parallel health system should be discouraged as this leads to reduced integration and potentially for less equitable healthcare access through a system which may not be sustainable [35].

Supporting Syrian HCWs to enter the workforce in Egypt and elsewhere is also cost effective compared to training doctors from undergraduate to specialisation [14]. Additionally, it will support Syrian and other refugee HCWs to earn, alleviating financial and associated mental health pressures.

### Strengths and limitations

Interviews for this research were undertaken in 2019 and it is likely that the situation in Egypt has changed since the COVID-19 pandemic. However, the findings remain pertinent and may not only have implications for refugee doctors in Egypt but also elsewhere given sparse literature on this topic. Snowball sampling was chosen to gain access to a population who are largely difficult to reach, in part due to their minority status in a large city, but also due to the low profile they maintain for personal security. However, this approach may have introduced a sampling bias and limited the findings only to those relevant to this particular group, and potentially missing out on information from those not affiliated with this group who may have offered alternative experiences. The consequence is that this group largely reflects Syrians who completed their medical education in Egypt, doctors over other healthcare professionals, younger KIs over older ones, and more men compared with women. There may have been other barriers faced by Syrian women in Egypt that the study did not explore sufficiently. We do not consider that the positionality of the interviewer as a man influenced the number of female participants, as the initial participant in the snowball sampling chain was known to the female authors prior to the study and all potential female Kis responded and were interviewed. It may rather reflect a potential impact of snowball sampling within Egypt and the socioeconomic pressures described by the female KIs. In addition to this, the field work was limited to Syrians living in Cairo, and experiences of Syrians in other cities such as Alexandria may have been different.

## Conclusion

To our knowledge, this is the first exploration of the experiences of Syrian refugee HCWs in Egypt. Though the Syrian KIs noted frustrations relating to education, societal changes, bureaucracy and finances, positive comments were made about the welcome from Egyptian society including medical professionals. Important considerations for the future relate to the introduction and enforcement of relevant policies which support Syrian HCWs to gain registration and official status in Egypt. This will have multilateral benefits, both for the refugees as well as the health system.

### Supplementary Information

**Additional file 1.**

## Data Availability

The datasets used and/or analysed during the current study are available from the corresponding author on reasonable request.

## Abbreviations

AUC: The American University in Cairo
CMV: Convention on the Protection of the Rights of All Migrant Workers and Members of their Families
ECHO: European Civil Protection and Humanitarian Aid Operations
EGSP: Egyptian Government Services Portal
EMRO: Regional Office for the Eastern Mediterranean
EMS: Egyptian Medical Syndicate
HCW(s): Healthcare worker(s)
IDP(s): Internally displaced person(s)
IRB: Institutional Review Board
KI(s): Key informant(s)
LSHTM: The London School of Hygiene and Tropical Medicine
MoHP: Ministry of Health and Population
NGO: Non-governmental organisation
ODK: Open Data Kit
PHR: Physicians for Human Rights
PRISMA: Preferred Reporting Items for Systematic Reviews and Meta-Analyses
RSDI: Refugee Status Determination Interview
UNHCR: United Nations High Commissioner for Refugees
WHO: World Health Organisation
