# Supplementary material for: Experiences of Egypt as a destination and transit country for Syrian refugee healthcare workers: a qualitative study
Source: BMC Health Serv Res. 2023 Aug 17;23:872. doi: 10.1186/s12913-023-09889-4 (PMC10433661; doi:10.1186/s12913-023-09889-4)
Supplement: Supplementary file 1 — Additional file 1. [file 12913_2023_9889_MOESM1_ESM.docx]

**Appendix 1: Interview guides**

Available in English and Arabic

**Introduction**

1. Please state the pseudonym you would like to use.
2. Where in Syria did you live?
3. How long have you lived in Egypt?
4. What is your current status in Egypt? (e.g. registered/non-registered)

**Work in Syria**

1. What is your healthcare discipline? (Examples: doctor, nurse, midwife, physiotherapist, pharmacist, dentist)
2. Where did you complete your training [Probe: Did you train in Syria? How long did you work there]?
3. What kinds of patients did you work with? Can you outline your experience?

**Moving to Egypt**

1. Can you tell me about the process of coming from Syria to Egypt?
2. Did you initially intend to keep working in your profession in Egypt? What did you know about the process prior to arriving?

**Work in Egypt**

1. Do you currently work in Egypt? What is your job? Have you tried to register as a healthcare professional in Egypt?
2. What were the challenges during this process? What do you feel worked well?

**Healthcare provision**

1. Have you provided healthcare services in Egypt? What kind of setting is this based in? Is this a public or private sector service? What groups of patients do you provide services to?
2. Are you remunerated for the service you provide? Does your work have conditions? Who regulates this?
3. Are there limitations to your practice while in Egypt? What are the main barriers?
4. What are your views about working in Egypt? Are there comparisons with working in Syria?
5. Do you have concerns about healthcare provision in Egypt, e.g. legality, informal care?

**General attitudes and recommendations**

1. What do you feel are people’s attitudes towards migrants working in healthcare?
2. Have there been any particular challenges when working in Egypt?
3. Do you feel that Egyptian health system provides adequate care for refugees at the moment? What could your experiences bring to improve this?
4. What could improve the process of registering and working in healthcare?
5. Do you intend to return to Syria to work in the future?
6. Any additional comments.

**Interview guide (government officials/other actors)**

**Introduction**

1. Please state the pseudonym you would like to be cited with.
2. What is your current role?

**Egyptian health system**

1. What is the current setup of the Egyptian health system? How is it primarily funded? What is the main route for people to access services?
2. What are the main burdens on the health system in Egypt at the moment?
3. Are there problems with recruitment of health professionals in Egypt? What have been the main barriers?
4. How do you think the Syrian refugee crisis has impacted the health system in Egypt? Can you make comparisons with other neighbouring countries?
5. What makes Egypt different to these countries?
6. Are there any specific problems you think need to be addressed with regards to this?

**Refugee employment**

1. What is the current process for registration as a refugee in Egypt? Are there different circumstances for Syrians?
2. What are the main existing policies regarding employment for refugees? Does this differ for refugees from Syria (or other Arab countries)?
3. What is the process for migrants becoming registered as professional healthcare workers in Egypt? Can refugees follow the same process? How long can they work for? What is the process for revalidation?
4. Are there any specific policies about refugees working in healthcare? What are general attitudes regarding this? Are there specific concerns?
5. What training is offered to refugees taking up work in healthcare? Who provides/funds this?
6. Does the involvement of any international bodies/NGOs help to facilitate this process?
7. What do you think are the main barriers to Syrians taking up employment in the health system? Are there specific conditions/requirements for them to work?

**General attitudes/recommendations**

1. What experiences do you think Syrians can bring to the Egyptian health system to improve it?
2. What do you think could be improved in the process for Syrians arriving in Egypt? Do you have any recommendations regarding the employment process?
3. Any additional comments.
